# New concept using Passive Infrared (PIR) technology for a contactless detection of breathing movement: a pilot study involving a cohort of 169 adult patients

**DOI:** 10.1007/s10877-013-9457-2

**Published:** 2013-04-03

**Authors:** V. Hers, D. Corbugy, I. Joslet, P. Hermant, J. Demarteau, B. Delhougne, G. Vandermoten, J. P. Hermanne

**Affiliations:** 1 Department of Pneumology, CHR Namur, 5000 Namur, Belgium; 2 WOW Technology, Engineering, 5000 Namur, Belgium; 3 School of Public Health, University of Liege, 4000 Liege, Belgium; 4 Department of Internal Medicine, CHR La Citadelle, 4000 Liege, Belgium; 5 Department of Internal Medicine, CHR Namur, 5000 Namur, Belgium; 6 2-Observe Company, 4500 Huy, Belgium

**Keywords:** Respiration monitoring, PIR technology, Apnea, Noncontact monitoring

## Abstract

A pilot study has been conducted to validate the Breath Motion Detecting System (BMDS), a new concept using Passive Infrared (PIR) technology for a contactless detection of respiratory movements. The primary objective of the study was to show if movements detected during sleep by the BMDS were indeed related to breathing. This medical device is not intended to measure the respiratory rate, but in a second step, it will be able to detect pathological central apnea in adults. One hundred and sixty-nine adult patients underwent a full polysomnography in which each respiratory movement was recorded concomitantly through the BMDS. Curves obtained by the BMDS were compared to those of thoracic movements recorded by classical piezoelectric belts and of pressure obtained with nasal cannula. The correlations between the PIR sensors were highly indicative of respiratory movement detection. Since PIR sensors are sensitive only to the exemplification of the rib cage, they did not detect obstructive apnea. Unfortunately, only a few patients in the studied population had a central apnea. Moreover as our sleep laboratory was equipped only with piezoelectric bands, the central apnea respiratory effort data are not a validated signal to be used during sleep recordings. The data recorded by the BMDS demonstrate the ability of the PIR technology to detect respiratory movements in adults. The concept is practical, inexpensive and safe for the patient. Further studies with respiratory inductive plethysmography are needed to investigate the potential of BMDS to detect central apneas.

## Introduction

Detecting and tracking the physical and physiological state of human beings is becoming a major focus of health research. Respiration is one of the vital signs indicating a person’s health status. Monitoring techniques are now mostly based on two different principles: the measurement of the respiratory effort (e.g. thoracic impedance pneumography, accelerometry, photoplethysmography) [1–4 **]** and the respiratory effect (e.g. sound recording, temperature sensing, carbon dioxide sensing) [5–7]. Thoracic impedance pneumography has been adopted as the gold standard in intensive care units (ICU) over the past decade, whereas in sleep studies, inductive plethysmography, often referred to as respiration band, is commonly used [8]. A tremendous amount of progress has been achieved in the monitoring and recording of vital signs, such as blood pressure, heart and breathing rate, over the past 30 years. All these technologies are generally found in intensive care or middle care environments. No technology has yet emerged to monitor vital signs, and this is particularly true in the case of individuals receiving nursing care in a hospital (out of ICU) and home medical care services. Nursing care for “borderline” patients with cardio-pulmonary or neurological disorders is always confined to measuring blood pressure, temperature, heart rate and sometimes oxygen saturation by pulse oximeter (just average 2–3 times per day). Subsequent to hospitalization in an ICU, the patient is usually no longer being monitored. The period immediately after the ICU can be critical in terms of vital complications. There is substantial evidence to show that changes in the respiratory rate can be used to predict potentially serious clinical events such as cardiac arrest or admission to the ICU. As described by Young et al. (2003), slow transfer to the ICU of physiologically defined high-risk hospitalized patients is associated with a higher risk of death. Slow response or no detection of physiologic deterioration may explain these findings [9].

In a bid to improve this situation, a pilot study has been conducted to validate the BMDS (Breath Motion Detecting System), a new concept using Passive Infrared (PIR) technology for a contactless detection of respiratory movements. Several new methods have recently been described using non-contacting techniques, such as the Doppler Effect [10, 11] ceiling-attached microwave antenna [12] and thermal infrared imaging for breathing monitoring [13, 14]. These techniques are being validated and most of them are not totally passive [15].

## Aims and objectives

The first goal of this pilot study was to show that movements detected during sleep by the BMDS were indeed related to respiration and subsequently that the BMDS is reliable for this purpose. The second aim was to determine the optimal positioning, angulation and distance for each PIR sensors to the patient in order to have fewer false negative detection of respiratory movement.

## Materials and methods

### Description of the device

A Passive Infrared sensor (PIR sensor) is a dual-element pyroelectric sensing device that reacts only to heat sources variation (such as the movement of the human body). All objects above absolute zero emit infrared radiation that remains invisible to the human eye but can be measured by electronic devices. When exposed to infrared radiation, the material of the pyroelectric sensor generates a surface electric charge. When the heat source is moving, the two elements do not receive the same amount of heat at the same time. By using a differential amplifier between both elements, it is possible to detect the difference in the amount of heat received and therefore the heat source motion. The term *passive* in this instance means that the PIR device does not emit an infrared beam but merely passively accepts incoming infrared radiation. Unlike their conventional use, the sensors are designed in this case for low amplitude movement detection such as chest and abdominal movement produced by respiratory effort or air temperature changes around the nose area. The sensors are sensitive to lateral motion but not to axial motion (in this case both elements receive the same amount of heat). As the patient’s position will change during sleep, more than one sensor must be used to ensure that breath movement can still be detected. The purpose of the device is to allow a simple, non-invasive, contactless, and instantaneous recording of respiratory movement. The first prototype of the BMDS included 8 PIR sensors placed around the chest (6 sensors) and the cephalic (2 sensors) of the patient. Signals are recorded on a laptop computer by means of an USB acquisition device. For the second prototype, we reduced the number of sensors to 6 based on the preliminary results. Signals are recorded with an autonomous system (AVR micro controller and SD card).

**Fig. 1:**
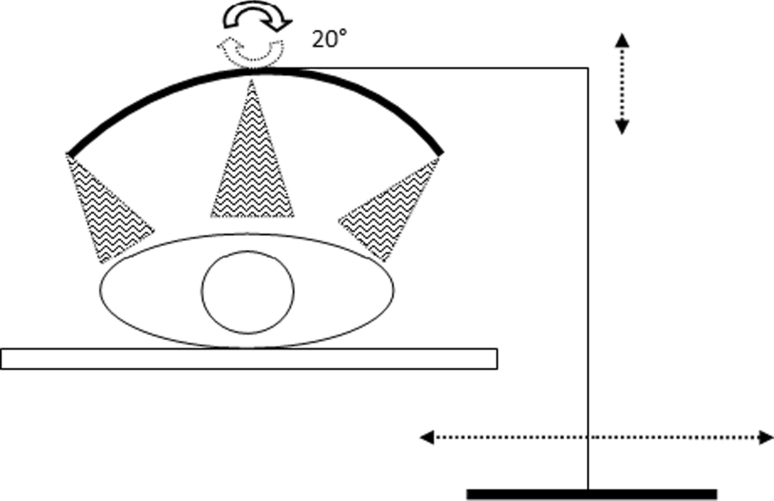
Three groups of 2 PIR’s captors side by side

And finally, the 3rd prototype consisted of six PIR sensors (3 groups of 2 captors side by side) in a semi-circle support around the patient’s chest (see Fig. 1). We therefore obtained 8 or 6 curves for each patient allowing an analysis with sensors from different positions.

### Data collection

The study was conducted in the Namur Regional Hospital (Belgium) sleep laboratory. One hundred and 69 subjects (aged 16 or older) were recruited among adult patients who underwent a full polysomnography during which each respiratory movement was recorded concomitantly via the BMDS. No patient was left out of this pilot study. The synchronization of the two recordings was performed by each device’s own internal clocks. Curves obtained via the BMDS were compared to the curves of thoracic movements recorded by conventional piezoelectric belts (Somnambule, Medatec^©^) [16], digital pulse oximetry (SpO2) and pressure curve obtained with nasal cannula [17]. All recording channels were calibrated according to the standard protocol followed in the sleep disorders centres by the same technician. The diagnosis of Sleep Apnea is based on the combination of the Apnea-Hypopnea Index (AHI), a clinical index measuring the average number of sleep disordered breathing (SDB) events per hour, and of a subjective measure of daytime sleepiness. According to a landmark study from the American Association of Sleep Medicine (AASM), it is estimated that 4 % of the male and 2 % of the female adult population are affected by sleep apnea [18]. It is important to note that the number of breathing events was especially sought in relation to a study to compare breath waveforms. Various parameters were correlated in analysis such as the patient’s gender, weight and height, body mass index (BMI), room temperature, covers, signals detected by the BMDS and polysomnography data converted into European Data Files (EDF) [19–21]. The type of bedclothes worn by the patient during recording was also studied. Three types of bedclothes were noted: shirtless, normal bedclothes and T-shirt. One adult volunteer was also recruited for apnea simulation (with T-Shirt).

### Data analysis

Data analysis was performed with algorithms designed by WOW technology. The operation of the signal on the basis solely of the amplitude is problematic because of the long periods of acquisition; the signal amplitude is comparable to the noise. On the other hand, the presence of a signal is easily detected by the presence of more or less regular oscillations (breathing is a quasi-periodic signal), whereas, the absence of signal is marked by rather chaotic oscillations, without regularity.

**Fig. 2:**
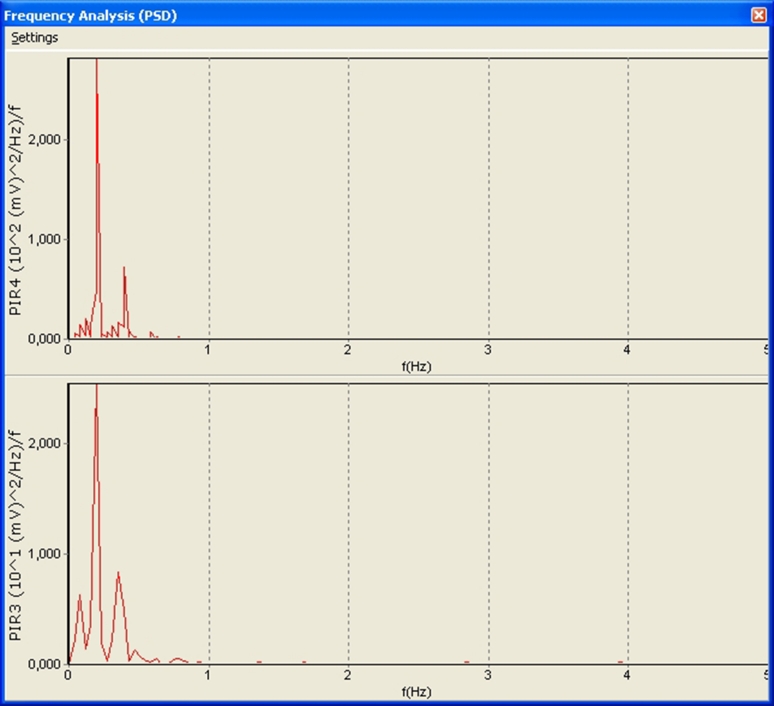
Spectral analysis before apnea

**Fig. 3:**
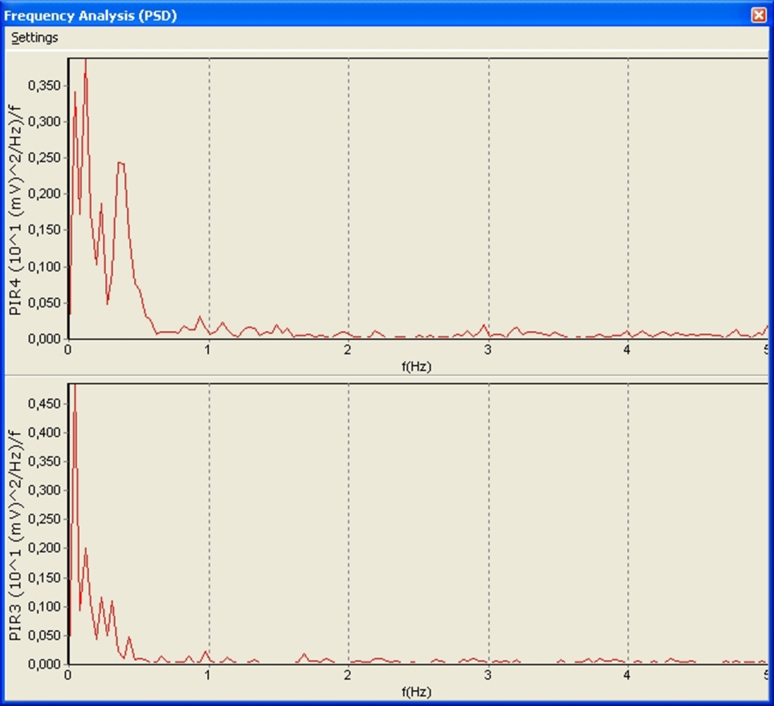
Spectral analysis during apnea

This can be demonstrated by the frequency analysis. With the presence of a regular signal, a peak frequency differs quite significantly in amplitude relative to the other peaks, whereas in the absence of a signal, peak amplitudes of these frequencies are much closer to each other (see Figs. 2, 3).

**Fig. 4:**
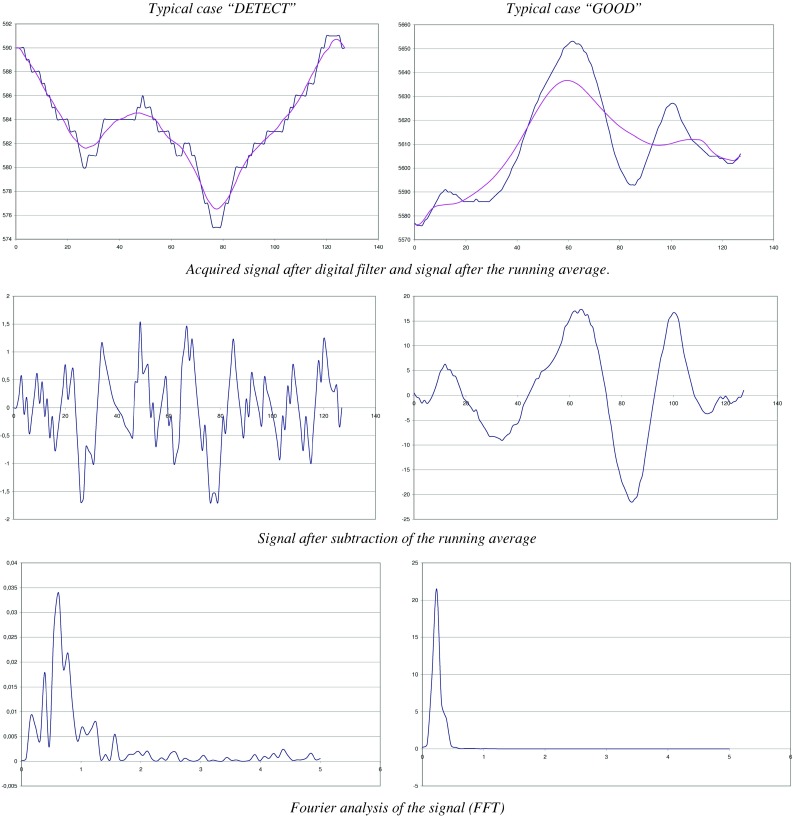
The main computation steps of our algorithm

Our algorithm is divided into several stages:

1. *Sampling* based on the pre-analyzes, we reduced the sampling frequency of 10 Hz. Previously, we were sampling 100 Hz (cut-off frequency = Fs/2 = 50 Hz), but the frequency band 5–50 Hz showed no or little signal. There was no reason to sample more than 10 Hz. The most beneficial effect of this change is to reduce by 10 the number of issues which considerably reduces the calculations.
2. *Digital Filter* for this signal, it is necessary to eliminate oscillations and peak amplitude too high. The peaks are removed by means of a median filter of 3 successive points. The aim of the filter is to replace the value of a point by the median value of this point and its two neighbours. For example, the values 12, 64, 13 have 13 as median. After removing peaks, applying a small low-pass filter to the data: each point is replaced by the average value calculated in the light of this point and its two neighbours. For example, the values 11, 11, 14 give an average of 12.
3. *Analysis window* we will try to characterize the signals every second. To do this, we need to analyze the signals during a time window that is not too short (if the characteristic quasi-periodic signal will not be useful) or too long (otherwise we could have a little of all cases shown in the window and be unable to discriminate sufficiently enough). As we will see later, we use spectral analysis, which, to allow the use of an algorithm of Fast Fourier Transform (FFT) [22], we have forced a number of points equal to a power of two in our window.
  Our choice was 128 points setting our window to a time of 12.8 s due to the sampling frequency (10 Hz or 10 points per second).
4. *Extraction of the signal’s useful component* over time, the signal shows a slow drift of significant amplitude compared to the useful signal. It is therefore necessary to highlight the useful signal by subtracting the DC component drift. This component is calculated by replacing each point in the window by a running average calculated with number of points (45 points in this case). This choice is justified by the fact that 45 points is 4.5 s which is a longer period than what is observed during normal breathing. For the points located at the ends of the window, the mean is simply calculated by a reduced number of points.
5. *Determination of average signal amplitude* a RMS (Root Mean Square) value of the signal is calculated on the window. This value is a measure of the oscillatory component of the signal.
6. *For spectral analysis* we calculate a FFT of the analysis window. We then determine the amplitude and frequency values for the two highest spectral signal peaks.
7. *Characterization of signal* each sensor signal is analysed individually.
  If the RMS amplitude of the signal is greater than ~625 mV, the sensor has detected a clearly significant movement. We classify the window as “MOVE”.
  If the RMS amplitude of the signal is greater than ~156 mV, regular oscillatory motion related to respiration is clearly identifiable. We classify the window as “GOOD”.
  Below this range, it becomes difficult to make a distinction between a signal and noise. So we use spectral analysis to characterize our window. If the main peak is outside the frequency range of 0.23–1.02 Hz, the characterization is automatically “DETECT” (no useful signal in the frequency range of plausible measure breathing and therefore suspected apnea). If the main peak is located in the frequency range of plausible the coefficient is calculated as follows (see Eq. 1) if the estimated coefficient is >10, it was a useful signal and the window enter the “GOOD” category. Otherwise, the window remains as “DETECT”.
    1 $$ \begin{aligned} & \left( {{\text{Peak1}} - {\text{Peak2}}} \right)^{2} /{\text{Peak2}}\,{\text{if}}\,{\text{Peak2}}! = 0 \\ & {\text{Peak1 if Peak2 }} = \, 0 \\ & {Peak1} = {amplitude of the largest peak} \\ & {Peak2} = {amplitude of the second largest peak}. \\ \end{aligned} $$
8. *Final result of the algorith*: The final result is obtained by considering the most favourable category of all signals (thus working with 6 sensors, we have 6 signals). If all signals simultaneously point to a suspected apnea (“DETECT” category), 20 successive windows (thus 20 s) with “DETECT” are awaited before declaring an apnea. The window is then reclassified as “BAD” (apnea or total loss of signal, these two cases are indistinguishable and could generate an alarm) (see Fig. 4).

## Results

A total of 169 patients aged 48.8 ± 12.0 years were engaged in this study. Eighty-seven subjects were recorded with the first prototype, 66 others with the second one, and the final 16 patients with the 3rd prototype. The group of subjects was heavier, with a mean body mass index of 29.9 ± 7.1 kg/m². The mean recording time was 07:36 h (range 03:00–08:30 h). Table 1 summarizes the demographics and patient characteristics.

**Table 1:** Baseline characteristics of 169 patients engaged in this study

Variable	Proportion (% or SD)
Gender	
Men	58 (34)
Women	111 (66)
Weight, Kg	89.03 (±20.2)
Height, cm	172.8 (±9.5)
BMI	29.9 (±7.1)
Room temperature, degree (°C)	22.6 (±3.3)
Mean recording time	07:36 (3–830 h)
Type of bedclothes	
Shirtless	0
Normal	89 (53)
T-shirt	80 (47)
Covers	
None	2 (1)
Bed sheet	7 (4)
Bed sheet + coverage	151 (90)
Quilt	9 (5)

Data are presented as mean ± SD, except gender, bedclothes and covers, which are reported as a number (%). *BMI* body mass index (kg/m²)

We observed a clear correlation between the different PIR sensors set around the patient for the detection of respiratory movement. The three generations of prototypes were different by the number and placement of PIR sensors. We have reduced the number of sensors from 8 to 6 and have removed the cephalic sensor that was the less performing one. The optimal position of the sets of PIR sensors is a disposition around the chest as shown above in Fig. 1. In a range of 20–50 cm between the patient and the PIR sensor, the recorded data showed quite comparable results.

**Fig. 5:**
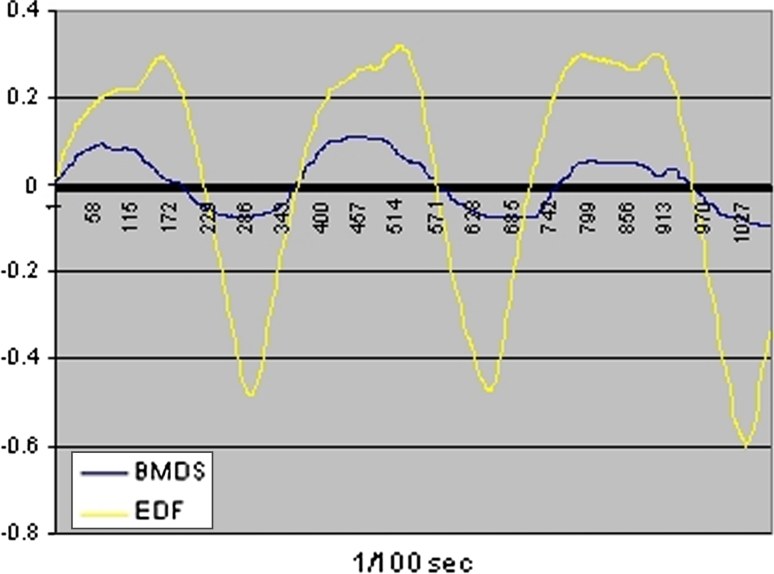
Periods when the BMDS’s signals are clearly oscillatory motion. There is a great deal of similarity between the measurements of both systems (BMDS and EDF). EDF* curve* represent the polysomnography data from piezoelectric bands of the recording thoracic respiratory effort converted into European Data Files. BMDS curve is recorded from one of 6 PIR sensors. Calculated frequency of 4 min = 0.3 Hz

Similarly, we found a high degree of concordance between the BMDS’s signals and conventional polysomnography sensors (see Fig. 5).

Each sensor’s degree of sensitivity in recording a movement is dependent upon the position. Data obtained by lateral sensors were more feasible. Our useful signal comes from detecting motion induced by the rib cage; hence it is strongly influenced by adjusting the position of the sensors. Moreover there was no correlation between PIR curves amplitudes and volume detection by piezoelectric bands.

Dependency between VTH and the received signal might have been assumed but the angle of the sensor is the decisive factor rather than the thoracic volume. No relationship is found between the patients’ gender, weight, height or BMI and the amplitude of the signals detected by the BMDS. Covers and room temperature have no significant impact on the useful signal received from the BMDS. When the patient is perfectly calm without any movement such as turning, agitation or REM sleep, the detection of a respiratory rate becomes possible but has not been investigated in this first study.

### Others movements by the patient

**Fig. 6:**
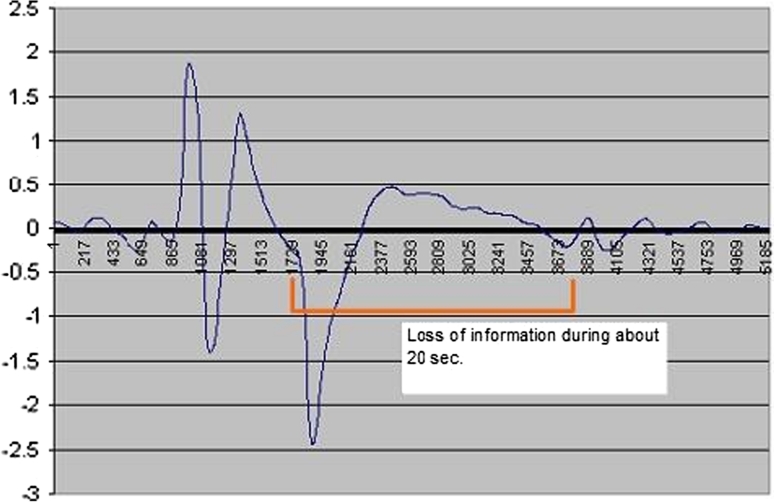
Recording period during significant movements of the patient. Blue* curve* is derived from PIR sensors

During the night, the patient moves and can be positioned on one side of the bed, sometimes with a blanket or a pillow that could occult some PIR sensors. So some sensors will not detect information while others will perceive the respiratory movements. Owing to the patient’s movements (turning, waking up, agitation) the sensors detect a lot of movement thus creating saturation. In this case information is lost on all the sensors for a period of 5–20 s (see Fig. 6) during which time the electronic device proceeds to desaturate the PIR sensors.

**Fig. 7:**
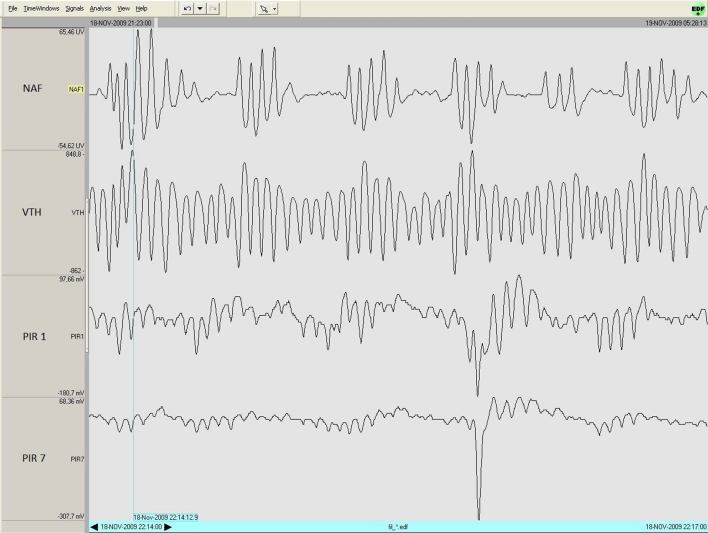
Obstructive apnea: *NAF* nasal airflow, *VTH* thoracic movements, *PIR* BMDS’s sensor signal. Complete screen shows a display time of 3 min

In our population of patients, obstructive apneas were more frequent than a central event. They are characterized by a blockage of the flow of air in the upper respiratory tract while thoracic movement is ongoing. Because the PIR sensor is sensitive only to the exemplification of the rib cage, it does not detect this kind of event. When the nasal cannula detects airflow interruption while the sensor of piezoelectric bands continues to perceive the movement of the chest, the BMDS signals are seen to do the same (see Fig. 7).

For the central apnea, unlike obstructive sleep apnea, the flow interruption is associated with a cessation of the thoracic and abdominal movement. Four patients had this type of apnea in the population we were studying. As our sleep laboratory was equipped only with piezoelectric bands, the data recorded for the central apneas are not presented in this study. In fact, the diagnostic accuracy must be validated by a respiratory inductive plethysmography (RIP) in a gold standard polysomnography (PSG).

**Fig. 8:**
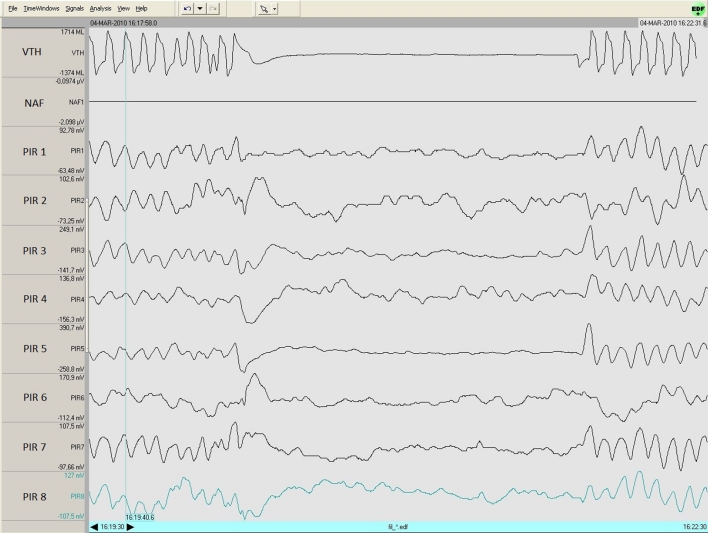
Simulation of central apnea by a volunteer: *NAF* nasal airflow, *VTH* thoracic movements, *PIR* BMDS’s sensor signal

For information, one volunteer subject who did not have any history of sleep disordered breathing has undergone several combined recordings with the BMDS and polysomnography. We once again observed a high degree of correlation in the different data (see Fig. 8).

Finally, we evaluated the sensitivity of the BMDS to detect respiratory movements of patients. This sensitivity is obtained by determining the number of false negative detection. We define it as an absence of respiratory movement recording while PSG piezoelectric bands would have record it (True Positive breathing/True Positive breathing + False Negative breathing). So if the episodes of central apnea are excluded from this study, the sensitivity of breath detection is very high, with a score of 98 %. However, compared with existing monitoring systems in hospitals (based on the placement of electrodes directly on the patient), this result is quite remarkable.

## Discussion

### Problem of positioning PIR sensors

This involved determining the position of the sensor offering optimum measurements of the movement made by the patient’s chest. The following observations were made: the closer sensors are to the chest, the more they are sensitive to the patient’s respiratory movement. The optimum position is one whereby the sensor is tipped towards the side of the patient. In this position it is easy to distinguish the patient’s breathing despite environmental disturbances. As sensors placed above the patient do not provide useful information, these sensors were discontinued in the 3rd prototype. The distance between the sensor and the patient has a significant impact on the measurements. Too wide a distance makes it difficult to distinguish the disturbances movements and breathing. The ideal distance appears to be 20–50 cm between the patient and the sensor. Logically, the degree of sensitivity decreases as the distance from the sensor increases. An analysis on a signal-by-signal basis, reveals there is often a lack of information for a period of 15–30 s in the case of some sensors. Fortunately, in most such cases, one or more others signals provided information at the same time. Periods when information is absent for more than 30 s are always “covered” by another signal.

### Problem of background noise

In the case of background noise, a recording made when the bed is unoccupied shows a very flat signal. This shows that in the absence of a patient, the sensors do not generate false signals that could be misinterpreted. It also shows that in the case of the patient’s total immobility, an alarm can be triggered without creating any ambiguity.

### Test measurement of respiratory rate

In phases where there is no movement that would saturate the sensor and where most of the signal consists of a simple respiratory movement of the rib cage, we tried to determine the respiratory rate. Overall, the results are too fragmented to be of any use. This is because it is quite difficult to achieve a correct measurement when the amplitude of the oscillation is relatively close to the amplitude of signal drift. The proof is provided by a Fourier analysis, showing that at times the dominant signal is the low frequency of drift and non-oscillation breathing. Some hardware upgrades may be able to produce a more stable signal.

### Miscellaneous

We still suspect that this technology will be effective for the detection of central apnea.

A second study is already planned to validate the detection of central apnea with the BMDS device in comparison with a gold standard polysomnography including RIP. The principle goal of this future study will be to validate BMDS as a device capable to accurately detect central respiratory arrest in a population of adult patients.

Noncontact respiration movement monitoring devices have many advantages over contact methods, especially for continuous supervision of the patient without causing any disturbance or imposing constraints on the patient’s actions and behaviour during the monitoring period. Looking to the future, the BMDS’s concept could monitor not only respiratory arrest, but also hyperactive movements of the patient (convulsions, psychomotor agitation, etc.) without electrodes, gel or sensors having to be used on the patient. Furthermore, the system is designed to be applied for individual continuous monitoring in general hospital wards and at home.

## Conclusions

The data recorded by the BMDS show the ability of the PIR technology to detect respiratory movement in adults. As far as we know, this research is the first one to explore the accuracy and feasibility of PIR technology in the context of detecting respiratory movement in hospitalized adult patients. This concept is simple, inexpensive and safe for the patient (no electromagnetic wave sent towards the patient). The potential benefit of performing this study is to provide a safe and effective technology for long-term monitoring of movement patterns in humans, which includes the significant physiological information relating to breathing as well as bodily motion. Further studies with respiratory inductive plethysmography are needed to investigate the potential of BMDS to detect central apneas for sudden death occurring in adults and in children.
